# Acute kidney injury associated with dengue virus infection: a review

**DOI:** 10.1590/2175-8239-JBN-2021-0221

**Published:** 2022-02-23

**Authors:** Paulo R Bignardi, Gabriela R Pinto, Maria Letícia N Boscarioli, Raissa A. A Lima, Vinícius D. A Delfino

**Affiliations:** 1 Pontifícia Universidade Católica do Paraná, Escola de Medicina, Londrina, PR, Brasil.; 2 Universidade Estadual de Londrina, Hospital Universitário, Departamento de Clínica Médica, Londrina, PR, Brasil.

**Keywords:** Dengue, Arbovirus infections, Acute kidney injury, Dengue virus, Dengue, Infecções Por Arbovírus, Injúria Renal Aguda, Vírus Da Dengue

## Abstract

Acute kidney injury (AKI) is one of the least studied complications of dengue, but it carries high mortality rates and prolonged hospital stay. Due to the severity of this complication, the risk of developing chronic kidney disease (CKD) and the increasing number of dengue cases reported worldwide, particularly in the tropical and subtropical regions of Africa, Southeast Asia and South America, including Brazil, we embarked on this narrative review, aimed to update the epidemiology of AKI associated with dengue, elucidate the main pathophysiological mechanisms of AKI caused by the dengue virus infection, as well as discuss useful information on the prevention and management of AKI in patients with dengue.

## Resumo

A injúria renal aguda (IRA) é uma das complicações da dengue menos estudadas, mas que acarreta altas taxas de mortalidade e prolongamento do tempo de internação. Devido à gravidade dessa complicação, ao risco de desenvolvimento de doença renal crônica (DRC) e ao número crescente de casos de dengue relatados no mundo, particularmente nas regiões tropicais e subtropicais da África, Sudeste Asiático e América do Sul, incluindo o Brasil, foi proposta esta revisão narrativa que objetivou atualizar a epidemiologia da IRA associada à dengue, elucidar os principais mecanismos fisiopatológicos da IRA causada pela infecção do vírus da dengue, assim como discutir informações úteis sobre a prevenção e o manejo da IRA em pacientes com dengue.

## Introduction

Dengue is the most widespread and rapidly growing vector-borne disease^1^. An estimated 2.5 billion people in 129 countries live in endemic tropical and subtropical regions and are at risk of contracting dengue, with 105 million people infected each year^1-4^.

In Brazil, the largest country in Latin America, dengue is an endemic infectious disease, with high morbidity and mortality rates, bringing a severe burden to healthcare services. In 2018, Brazil registered 265,934 cases of dengue, against 1,544,987 cases in 2019. In 2020, there were more 987,173 cases registered, with 554 confirmed deaths^5-7^.

Dengue is a viral infection transmitted by arthropods, whose vector is the female mosquito of the genus Aedes, mainly Aedes aegypt^8^. This infection is caused by an RNA virus of the Flaviviridae family, which has different serotypes: DENV-1, DENV-2, DENV-3 and DENV-4^9-11^. Recently, the existence of a fifth serotype (DENV-5) of isolates in Malaysia was postulated during an outbreak in 2007^12,13^. However, the scientific community is still waiting for the documentation of new isolates of this serotype^5^ to confirm whether or not it is a new dengue serotype or another unidentified arbovirus^14^.

This illness can range from subclinical illness to flu-like symptoms and, although less common, some patients develop the severe form of the disease, with severe bleeding, organ involvement and/or plasma leakage with reduced blood volume. It can be classified as: dengue without warning signs, dengue with warning signs and severe dengue^15,16^. One of the complications of dengue, associated with high morbidity and mortality rates, is acute kidney injury (AKI)^17^.

The pathophysiological mechanisms of kidney injury caused by the dengue virus are still not fully understood, with gaps in understanding and, consequently, in the prevention and adequate management of this serious complication in patients with dengue. Thus, the objective of this narrative review is to update the epidemiology of AKI associated with dengue, as well as to review the possible pathophysiological mechanisms of this lesion and to discuss the recommendations for the management and prevention of AKI in patients with dengue.

## Epidemiology of kidney injury in dengue

AKI, an abrupt decrease in the glomerular filtration rate, is one of the serious complications found in dengue. In recent decades, the incidence of AKI associated with dengue virus infection has increased significantly^10^.

**Figure 1:**
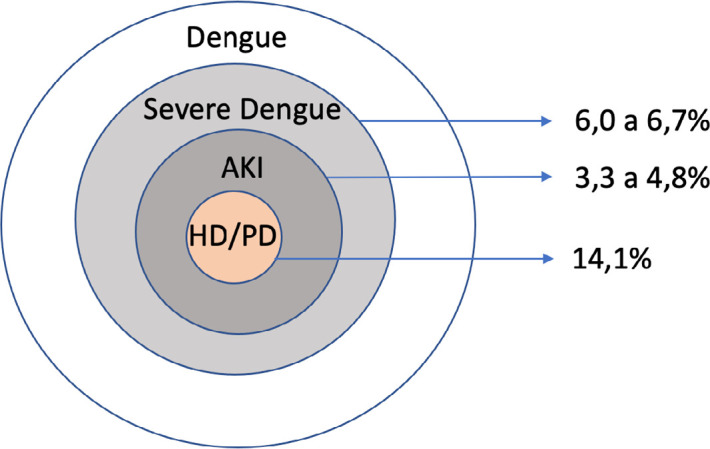
Estimate of AKI incidence in patients with dengue. PD: peritoneal dialysis; HD: hemodialysis; AKI: Acute Kidney Injury.

Severe dengue affects about 6.0 to 6.7% of patients diagnosed with dengue^17,18^. Among hospitalized patients with severe dengue 3.3% to 4.8% develop AKI, of which 14.1% require dialysis^17,19-24^. The need for dialysis can reach 70% in patients with dengue in intensive care units (ICU)^23^. AKI is associated with increased length of hospital stay and fatal cases of dengue^17,19^. Figure 1 shows the estimated incidence of AKI in patients with dengue.

Independent risk factors for the development of AKI in patients with dengue are advanced age, male gender, obesity, hemorrhagic fever, rhabdomyolysis, multiple organ dysfunction, diabetes mellitus, concomitant bacterial infection, delay in hospital consultation and use of nephrotoxic drugs^17,23,25,26^.

Thomas et al. studied the impact on the renal function of patients hospitalized with dengue^27^. The study showed that 73% of patients with non-dialysis chronic kidney disease (CKD) required hemodialysis, against 8% in the kidney transplant group and none in the control group, formed by patients with normal kidney function. After 2 weeks, 32% of patients in the CKD group were still dependent on hemodialysis against none in the other two groups, showing the role of dengue in renal impairment and the high percentage of acute-on-chronic kidney disease, signaling the possibility of definitive renal worsening in some of the affected patients.

## Pathophysiology of ira associated with dengue

**Figure 2:**
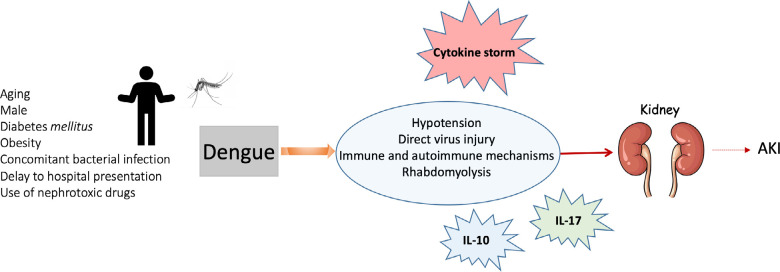
Risk factors and pathophysiological mechanisms involved in the development of dengue-induced AKI.

The pathophysiological mechanisms of kidney injury by the dengue virus are still not completely clear, but several hypotheses can be considered, including shock mechanisms resulting from hypotension, direct injury caused by the virus, indirect mechanism via the immune system and rhabdomyolysis. The combination of two or more of these mechanisms has yet to be considered. Figure 2 shows the associated risk factors and possible mechanisms involved in the development of dengue-induced AKI.

Kidney injury in dengue is likely due to the hemodynamic fluctuation that can occur throughout the clinical course of the disease. A number of cytokines are associated with dengue severity, including TNFα, interleukin-6 (IL-6), interleukin-8 (IL-8), interleukin-10 (IL-10), interleukin-12 (IL-12), interleukin-17 (IL-17), macrophage migration inhibition factor, high mobility group box 1 (HMGB1), monocyte chemo-attractant protein-1 (MCP-1) and matrix metalloproteinases^10,28-31^. This cytokine storm, in addition to activating the complement system and endothelial damage, results in increased vascular permeability with consequent hemoconcentration^28,32^. This process can result in shock, leading to reduced renal perfusion and kidney injury^28,33^. Shock or hypotension is present in 16% to 100% of patients who develop dengue-induced AKI^34,35^. Endothelial damage directly caused by the virus can also change vascular permeability, aggravating the hemodynamic instability^36^.

Although these mechanisms that cause hemodynamic instability are well established with regards to AKI-induction in dengue, the absence of shock and hypotension in a subgroup of patients with AKI in severe dengue points to the involvement of other mechanisms of injury which are discussed below.

A recent study compared the levels of cytokines in the acute phase of dengue and COVID-19, and found high levels of several cytokines throughout the course of these diseases, including high levels of immunosuppressive cytokines such as IL-10. Elevated IL-10 levels during the onset of viral infections can likely result in an inadequate antiviral immune response, which can lead to disease severity^33^.

Dengue virus appears to have broad cell tropism, replicating in hepatocytes, type II pneumocytes and cardiac fibers, as well as resident and circulating monocytes/macrophages and endothelial cells^36^. A study with histopathological analysis after death from dengue showed microabscesses in the heart, lungs, brain and kidneys^37^.

Added to this, the presence of viral particles in the kidney tissue after the death of a patient with dengue, revealed in a study that applied the immunological technique, supports the hypothesis of possible direct kidney damage^38^. More recently, evidence has emerged that the dengue virus, when infecting the kidney tissue, may be associated with the development of glomerulopathies, such as, for example, focal and segmental glomerulosclerosis^39^.

Another hypothesis of kidney injury induced by dengue is immune complex-mediated glomerular damage. Glomerular deposits of IgG, IgM and C3 were found in patients with dengue-induced renal failure^40^. After injecting the dengue virus in adult mice, Boonpucknavig et al. found proliferating glomerular lesions in the second week, and there were immune complexes in the third week^41^.

This issue is not definitively settled. For instance, Wiwanitkit commenting on studies carried out by himself and other researchers on the characteristics of the derived dengue virus-immunoglobulin immune complex considered It unlikely that immune complexes play a significative role in the pathogenesis of the AKI associated with dengue infection. Further studies in this field are therefore needed^42,43^.

On the other hand, some studies involving IL-17 may reinforce the role of the autoimmune response in severe dengue as a cause of AKI. Cytokines are needed to trigger the inflammatory response in host defense. IL-17 appears to play a significant role in immune-mediated glomerular diseases^44^. Additionally, the role of IL-17 in the pathogenesis of dengue has been studied. Jain et al. associated high levels of IL-17 with severe dengue^31^. Recently, a study in mice with ischemia-induced AKI demonstrated that the neutralization of some cytokine members of the IL-17 family attenuated tubular damage, renal oxidative stress and renal inflammation^45^.

Rhabdomyolysis, characterized by the extravasation of muscle content, including electrolytes, myoglobin and other muscle proteins, has also been described as a mechanism for AKI development. It is estimated that 13-50% of patients with rhabdomyolysis by any cause have AKI^46^. Muscle biopsies from patients with rhabdomyolysis reveal muscle abnormalities such as inflammatory infiltrates and myonecrosis. In dengue, rhabdomyolysis is mediated by direct viral muscle invasion or by myotoxic cytokines, which can cause AKI by deposition of myoglobin along the renal tubules and its precipitation after interacting with the Tamm-Horsfall protein in the presence of acidic urine, leading to tubular obstruction. Myoglobin can also lead to tubular damage and intrarenal vasoconstriction^46,47^.

AKI by hemolytic uremic syndrome (HUS) has been described in patients with severe dengue This clinical situation is composed of hemolytic anemia, thrombocytopenia and AKI^48^. Histopathological analyzes of patients with HUS revealed the presence of glomerular microthrombi, confirming renal impairment in these individuals^49^.

## Management of dengue-induced AKI

Dengue patients who develop AKI need longer hospital stay (an increase of, on average, 3 days) and there is higher mortality among patients with severe dengue-induced AKI^26^. This shows that AKI plays an important role in the prognosis of patients with dengue. Although the aspects of longer hospital stay and mortality point to a worse prognosis for the patient, the specific treatment for dengue-induced AKI is still limited and not very well established.

Patient assessment in the case of warning signs of severe dengue fever together with blood volume assessment are important starting points for the prevention of AKI in dengue patients. Fluid administration should be optimized (preferably crystalloid) with infusion rates and controlled tonicities, to avoid osmolarity disorders, fluid overload and worsening of intravascular fluid extravasation^50^.

The maintenance of electrolyte balance should be prioritized, due to the high prevalence of hyponatremia in these patients, and it is important that fluid therapy bear a higher tonicity, if such electrolyte abnormality is detected. Monitoring serum creatine phosphokinase (CPK) levels is important for early detection of rhabdomyolysis^48,50^. The use of steroids in patients with dengue is still controversial, and there is no indication for their use for AKI prevention in patients with dengue^48,51,52^.

Finally, renal replacement therapy may be necessary in some patients with AKI, especially in the presence of refractory uremia and hypercatabolism, metabolic acidosis, hyperkalemia and hypervolemia. In unstable patients with dengue hemorrhagic fever and AKI undergoing dialysis treatment, continuous hemodialysis has been recommended in those services where it is available, with the suggestion that peritoneal dialysis is associated with worse outcomes^35,50,53^.

The prompt and careful medical evaluation of patients with severe dengue is very important, with maintenance of blood volume, avoiding the use of nephrotoxic drugs, with clinical and laboratory monitoring for early identification of AKI to enable better management of the condition and avoid its complications.

We emphasize the need for post-discharge follow-up of individuals who developed AKI in dengue. It is known that patients with acute renal involvement, without previous CKD, can progress to CKD. In cases of individuals with acute-on-chronic kidney disease associated with dengue, progression to more advanced stages of the disease may occur^54,55^.
